# Fast and fully-scalable synthesis of reduced graphene oxide

**DOI:** 10.1038/srep10160

**Published:** 2015-05-15

**Authors:** Sina Abdolhosseinzadeh, Hamed Asgharzadeh, Hyoung Seop Kim

**Affiliations:** 1 Department of Materials Engineering, University of Tabriz, Tabriz 51666-16471, Iran; 2 Department of Materials Science and Engineering, Pohang University of Science and Technology (POSTECH), Pohang 790-784, South Korea

## Abstract

Exfoliation of graphite is a promising approach for large-scale production of graphene. Oxidation of graphite effectively facilitates the exfoliation process, yet necessitates several lengthy washing and reduction processes to convert the exfoliated graphite oxide (graphene oxide, GO) to reduced graphene oxide (RGO). Although filtration, centrifugation and dialysis have been frequently used in the washing stage, none of them is favorable for large-scale production. Here, we report the synthesis of RGO by sonication-assisted oxidation of graphite in a solution of potassium permanganate and concentrated sulfuric acid followed by reduction with ascorbic acid prior to any washing processes. GO loses its hydrophilicity during the reduction stage which facilitates the washing step and reduces the time required for production of RGO. Furthermore, simultaneous oxidation and exfoliation significantly enhance the yield of few-layer GO. We hope this one-pot and fully-scalable protocol paves the road toward out of lab applications of graphene.

Graphene, a monolayer of sp^2^-bonded carbon atoms has stimulated vast scientific interest due to its unique properties; enormous specific surface area (2620 m^2^ g^−1^), excellent mechanical properties (Young’s modulus of 1 TPa and intrinsic strength of 130 GPa), very high electronic conductivity (room-temperature electron mobility of 2.5 × 105 cm^2^ V^−1^ s^−1^), exceptionally high thermal conductivity (above 3000 W m K^−1^), along with many other outstanding properties have made graphene an interesting material for various applications^1^,^2^. Drug delivery^3^, hydrogen storage^4^, fuel cells^5^, supercapacitors^6^, batteries^7^, solar cells^8^,electromagnetic shields^9^, reinforcement for polymer, ceramic and metal matrix composites^10^,^11^,^12^, as well as conductive paints and inks^1^ are just a few of its countless applications. Nevertheless, the commercial applications of graphene are still limited due to the high cost and the low capacity of current production methods.

To date, the most common approaches for the large-scale synthesis of graphene have been based on exfoliation of graphite. The major differences between them are the yield and the defect content of their products. Although the use of less-defective graphene is essential in some applications (e.g. electronic devices), in some other cases, the defects and imperfections of graphene are less important or even desirable^9^,^13^. The oxidative-exfoliation methods can potentially produce large quantities of graphene oxide (GO), a graphene-like nanosheet which is typically defective and requires additional treatments to reduce it to reduced graphene oxide (RGO)^14^. The attachment of the oxygen-containing functional groups (OFGs) during the oxidation process increases the distance between graphitic layers^15^, hence weakening the van der Waals forces and facilitating the exfoliation. In order to remove oxidizing agents as well as other impurities from graphite oxide, several washing steps are required prior to the exfoliation. Washing the graphite oxide, especially in a large-scale, by means of conventional methods such as filtration^15^, centrifugation^16^ or dialysis^17^ is a real nightmare! Filtration processes are extremely time-consuming since graphite oxide particles (especially exfoliated ones) rapidly clog the filter pores. Moreover, high-speed centrifuge systems are less common in industrial applications due to their limited capacities and high costs. On the other hand, the dispersibility of GO increases by the progression of washing steps^18^. Regardless of the oxidation process, reduction adds yet another step to the synthesis procedure, prolonging the overall production time.

Due to the lack of a reliable method to supply the large demand for graphene, we have developed a fast and fully-scalable method for mass production of RGO. The consecutive stages of RGO production are shown in Fig. 1. The oxidation of graphite was adapted from Hummers method with some modifications; graphite powder reacted with potassium permanganate in a concentrated sulfuric acid solution, while the suspension was sonicated for specific periods of time [see Methods]. Considering that a graphite particle is a stack of several thousand graphene single layers, although few outer layers are easily oxidized during the oxidation process, complete oxidation of the inner layers takes longer. We believe that ultrasonic treatment of graphite during oxidizing can peel apart the oxidized layers and expose the inner layers to the chemical oxidants. Although the sonication-assisted oxidation of graphite considerably enhances the oxidation efficiency, its use in typical oxidation-based processes is impractical since the subsequent washing of exfoliated graphite oxide is virtually impossible. L-ascorbic acid as a potent reducing agent serves double functions in the present method: reduction of hydrophilic GO to hydrophobic RGO and conversion of residual Mn (VII) ions to soluble Mn (II) ions. Interestingly, the resultant RGO is easily filterable even by cellulose filter papers, whereas other scalable washing techniques such as ‘decanting and washing’ are also practicable due to the great tendency of RGO to settle down in the washing solution.

The main intent of this article is to propose a general approach for large-scale production of RGO so that the main idea, reduction of GO prior to washing, can be extended to most of oxidation-based synthesis methods of RGO. Ascorbic acid as a green alternative for conventional reducing agents of GO (e.g. hydrazine hydrate) is an inexpensive and abundant substance which can reduce GO to an acceptable extent^19^. The comparison of the reduction efficiency of GO suspensions by different reducing agents (sodium borohydride, pyrogallol, ascorbic acid, and hydrazine) revealed that only ascorbic acid could reduce GO suspensions to a level that is comparable with that of hydrazine^20^. Not only ascorbic acid but also any other reducing agents which preserve their functionality in acidic or near-neutral pHs can be employed in this method as well. This is due to the fact that the adjusting of the pH to the alkaline pHs will result in the formation of manganese or other metallic precipitates. In this regard, ascorbic acid is preferred to the conventional reducing agents since their best functionalities are in alkaline pHs.

## Results and discussion

**Figure 2:**
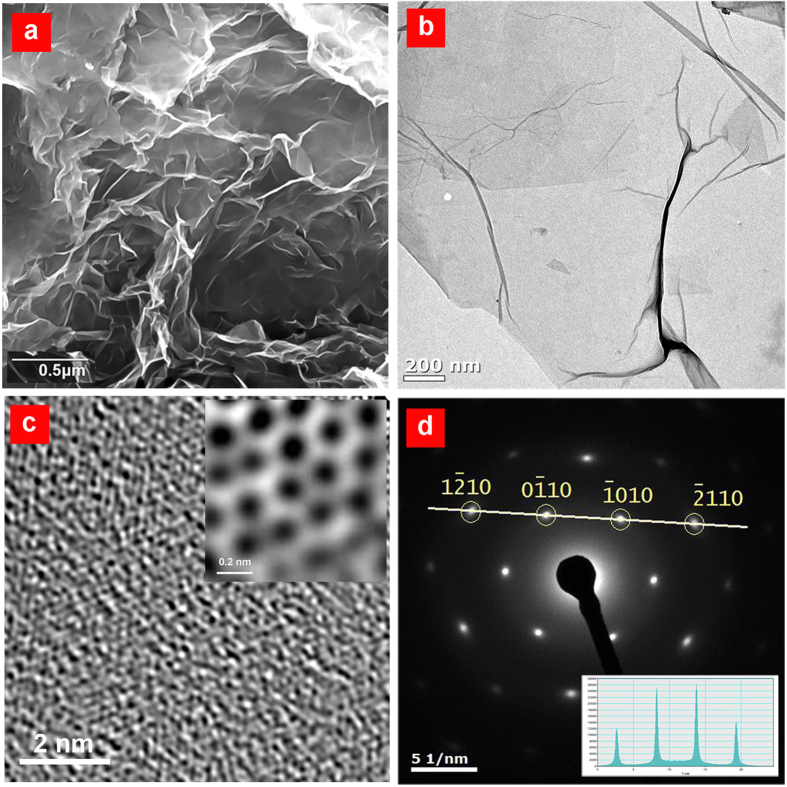
Electron microscopy imaging of ARGO powder. **a** SEM image of fully exfoliated and agglomerated sheets without gold coating. **b** TEM image of a few-layer ARGO. **c** HR-TEM image of a single layer ARGO. The color-inverted HR-TEM image after correction with Gatan microscopy suite software is shown as an inset. **d** SAED pattern of the ARGO shown in **b** with corresponding Miller-Bravais indices. The color intensity profile of the line passing through (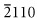) and (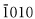) spots are shown as an inset.

The synthesized RGO (ARGO) was characterized by various chemical and physical methods. A sample was also taken from the oxidized graphite (AGO) prior to the reduction step with a great exertion for washing [see Methods]. The morphology and microstructure of ARGO sheets were investigated using field emission scanning electron microscopy (FE-SEM) and transmission electron microscopy (TEM) techniques. Well-exfoliated but crumpled and aggregated RGO sheets are observable in the SEM image (Fig. 2a), which is a common feature of the oxidation/reduction based methods^15^,^21^. The ARGO mostly consists of single- and few-layer sheets. By employing the edge counting method in TEM images taken from several flakes, the number of layers was determined to be no more than five. Representative TEM and high-resolution TEM (HR-TEM) images from a single-layer RGO are shown in Figs. 2b,c. The selected area electron diffraction (SAED; Fig. 2d) illustrates a symmetric six-fold pattern with corresponding Miller-Bravais indices which refers to graphite/graphene. Previous computational and experimental investigations^22^,^23^ suggest more intense spots on SAED pattern for {2110} planes relative to {1100} planes in a multi-layer AB stacked crystal. However, for a single-layer structure, a higher relative intensity of {1100} to {2110} spots is expected (I_{1100}_/I_{2110}_ > 1). Diffracted intensity profile along the passing line from (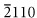) and (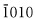) (shown as an inset in Fig. 2d) exhibits I_{1100}_/I_{2110}_ ≈ 1.96, confirming the single-layer structure of ARGO.

**Figure 3:**
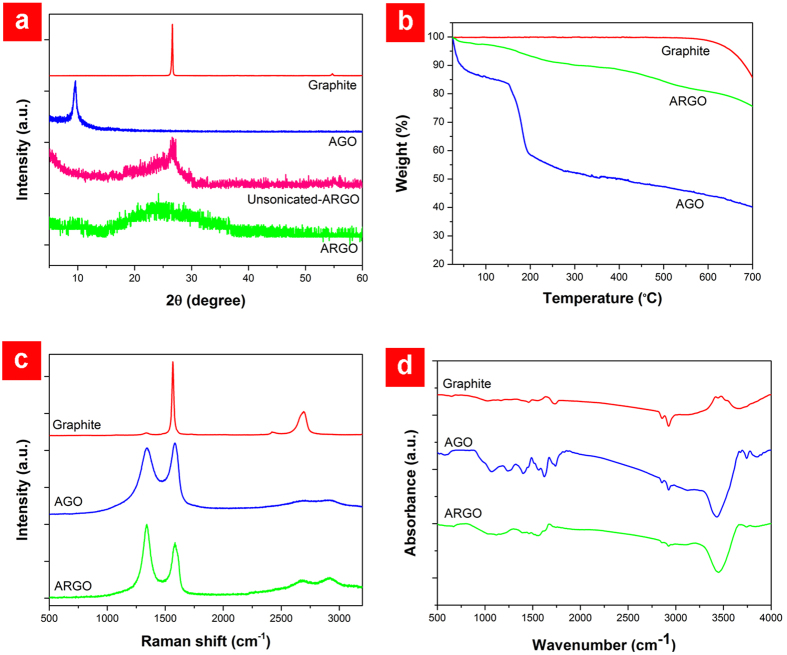
Characterization of ARGO powder. **a** Normalized XRD patterns of graphite, AGO, ARGO and unsonicated ARGO. **b** Thermal behavior of graphite, AGO and ARGO under air with a ramp rate of 1 °C min^−1^. **c** Normalized Raman spectra of graphite, AGO and ARGO excited by a 532 nm laser. **d** FT-IR spectra of graphite, AGO and ARGO.

X-ray diffraction (XRD) patterns of pristine graphite, AGO, and ARGO are represented in Fig. 3a. It is well established that the main graphitic peak at 2θ = 26.7° (corresponding to interlayer spacing of 3.34 Å) gradually disappears by the advancement of the oxidation process while a new peak appears at a lower angle (2θ ≈ 11°). The attachment of OFGs increases the interlayer spacing of graphite; thus, the position of this new peak mainly depends on the oxidation degree of graphite^24^. Here, AGO exhibits a single peak at 2θ = 9.5° which refers to an interlayer spacing of 9.25 Å. Reduction of GO leaves aggregated and randomly packed RGO sheets with a broad and low intensity XRD peak centered at 2θ ≈ 25°. To investigate the role of sonication on oxidation, a sample was synthesized by the same method employed in the preparation of ARGO but without ultrasonic treatment during the oxidation stage (unsonicated ARGO). Detection of the low-intensity graphitic peak in the XRD pattern of unsonicated ARGO indicates incomplete oxidation of graphite. The presence of remaining graphite after the oxidation stage has been frequently reported in conventional oxidizing protocols^15^. Nevertheless, sonication-assisted oxidation of graphite effectively resolved this problem so that no graphitic peaks were detected in the XRD pattern of ARGO. It should be noted that excessive ultrasonic treatment may result in the fragmentation of graphene layers^23^, hence care should be taken to avoid high power and prolonged sonications.

Thermogravimetric analysis (TGA, Fig. 3b) demonstrates that graphite is stable in air below 600 °C. A significant weight loss (~13%) occurred below 100 °C in AGO which is attributed to the evaporation of adsorbed water molecules due to the hydrophilic nature of GO. The notable weight decrease (~30%) between 150 °C and 195 °C is resulted from decomposition of OFGs to H_2_O, CO_2_, and CO gases. The weight loss with a slower rate at >300 °C is assigned to the removal of more stable OFGs^15^. The TGA analysis of ARGO depicts a continuous weight loss with a relatively constant rate, revealing a higher stability compared to AGO. At 700 °C, the ARGO exhibits a much smaller weight loss (~25%) than AGO (~60%) but still higher than graphite (~15%).

Raman spectroscopy was used to monitor structural changes during the oxidation and reduction processes. As shown in Fig. 3c, pristine graphite has a prominent peak at 1566 cm^−1^ (G band) which is due to the first-order scattering of the E_2g_ mode and two weaker peaks located at 1340 cm^−1^ and 2687 cm^−1^ (D and 2D bands, respectively) originated from the second-order double resonant process between non-equivalent K points in the Brillouin zone of graphene^25^. Structural defects induced by attachment of OFGs to the basal plane of graphene during the oxidation of graphite significantly intensified the D band and up-shifted the G band to 1585 cm^−1^ in the Raman spectrum of AGO. The blue shift of the G band has been attributed to the activation and merging of the Raman-inactive D′ band with the G band, the exfoliation of graphite to a single graphene sheet, and the resonation of isolated double bonds at higher frequencies than the G band of graphite^26^. On the other hand, the red shift of the G band in the Raman spectrum of ARGO to 1579 cm^−1^ was observed that can be ascribed to the reduction of AGO. Such a shift back has been reported in both chemical and thermal reductions of GO^2^,^26^. The relative intensity of the D and G bands (I_D_/I_G_), which is an estimation of the disorder level in graphene, exhibits a non-monotonic behavior. In a low concentration of defects, the introduction of new defects creates more elastic scattering, resulting in an increase of I_D_/I_G_. Further addition of defects leads to a more amorphous structure, attenuating all Raman peaks and decreasing I_D_/I_G_ (ref. 27). Consequently, by reducing the highly defective AGO (to ARGO), the relative intensity of the D to G band increased from 0.95 to 1.33 due to the elimination of defects (e.g. OFGs).

Since the attachment of the OFGs to the graphite layers during the oxidation dramatically affects the structure and properties of graphene, their removal is a great concern for a successful RGO production. Although numerous models for the chemical structure of graphene oxide have been proposed to date, there is still a considerable vagueness. Nevertheless, it is widely accepted that the hydroxyl and epoxide groups attached to the basal plane of the graphene and carboxyl and carbonyl groups located at the edges are the dominant functional groups^21^,^28^,^29^. Addition and elimination of these groups during the oxidation and reduction stages were investigated by Fourier transform infrared spectroscopy (FT-IR) and X-ray photoelectron spectroscopy (XPS). Regarding the FT-IR spectra (Fig. 3d), the broad signal between 3200 cm^−1^ and 3700 cm^−1^ is generated from O–H stretching vibration and adsorbed water molecules. Two peaks at 2925 cm^−1^ and 2855 cm^-1^ are assigned to asymmetric and symmetric vibrations of CH_2_ groups, respectively. The strong signal at 1738 cm^-1^ in the AGO’s spectrum refers to the C=O stretching vibration which was significantly weakened after reduction (ARGO’s spectrum). The peak at 1622 cm^-1^ is attributed to the C = C stretching vibration. Other prominent signals in the AGO’s spectrum which have been attenuated in the ARGO’s spectrum are 1399 cm^-1^, 1240 cm^−1^, and 1071 cm^-1^ originating from the O–H deformation, the C–O (epoxy) stretching vibration, and the C–O (alkoxy) stretching, respectively^19^.

**Figure 4:**
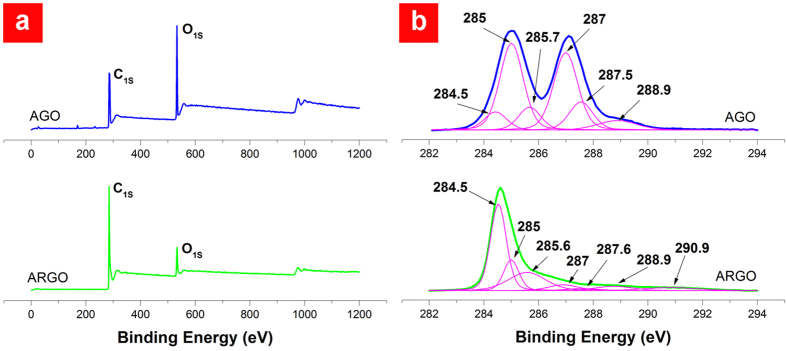
X-ray photoelectron spectroscopy of ARGO powder. **a** XPS survey spectra of AGO and ARGO. **b** Deconvoluted C_1S_ signals of AGO and ARGO.

The oxygen content of RGO mostly expressed as carbon to oxygen ratio (C/O) is a widely accepted criterion for evaluation of the reduction process. According to the XPS survey spectra (Fig. 4a), the C/O ratio has increased from 2.4 in AGO to 8 in ARGO, verifying a successful reduction process. After performing the Shirley background correction, the high resolution C_1S_ peaks were deconvoluted using Gaussian-Lorentzian functions (Fig. 4b). The assignments of the deconvoluted components were based on theoretical predictions of core level shifts and on reported spectra containing the particular oxygen functional groups^30^. The main peaks located at 284.5 eV and 285 eV are assigned to the sp^2^ and sp^3^ hybridizations of carbon atoms, respectively. The majority of carbon atoms in AGO has the sp^3^ hybridization due to the formation of covalent bonds during the oxidation stage while sp^2^ component originates from unoxidized carbon atoms. Although the reduction process recovers most of the sp^2^ bonds, some carbon atoms remain in sp^3^ hybridization. It is noteworthy that a fraction of sp^3^ bonds refers to the covalent C–H bonds, which is in agreement with CH_2_ groups detected in the FT-IR spectra (Fig. 4b). The chemical shifts of the core level to higher binding energies are attributed to the attachment of more electronegative OFGs. It should be noted that the values of these shifts might have some deviations^31^. The components located at 285.7, 287, 287.5, and 288.9 eV result from four main OFGs, namely hydroxyl (C–OH), epoxide (C–O–C), carbonyl (>C=O), and carboxyl (O=C–OH) groups, respectively. The 290.9 eV component in ARGO is originated from the shake-up satellite peak of sp^2^ bonds^30^.

As an important characteristic of the synthesis method, the powder yield was determined by performing a complete process, starting with 5 g graphite and obtaining ~6.37 g ARGO powder, which indicated an extremely high powder yield of ~127%. The yield of higher than 100% is attributed to the remaining of some functional groups as described earlier. In order to evaluate the purity of the final product, the metallic ion contents of the ARGO, especially Mn, K, and Na ions which had been introduced to the system during the synthesis procedure were determined using inductively coupled plasma optical emission spectrometry (ICP-OES) and the results are given in Table. 1. The metallic ion contents of the starting graphite were significantly decreased after oxidation step (in AGO) while a better removal of ions occurred after reduction process (in ARGO). The higher purity of ARGO is ascribed to the capability of ascorbic acid in reduction of insoluble ions to soluble species and to the superior and facile washability of ARGO.

In summary, the oxidation of graphite significantly increases its interlayer spacing and effectively facilitates the exfoliation process, but necessitates various post-treatments to remove the chemical oxidants which extremely prolongs the production time. While the use of filtration and centrifuge systems in typical oxidation-based methods are indispensable, they limit the production yield to few grams a day! Although the main idea behind the present method is simple, it removes a huge obstacle from conventional synthesis methods of graphene. GO loses its hydrophilicity which makes the whole process up-scalable and one-pot oxidation-exfoliation-reduction process, considerably reduces the overall production time from several days to few hours, hence kilogram or even ton scale production of RGO is simply attainable just by increasing the capacity of the reaction vessels. The SEM, HRTEM and SAED analyses clearly demonstrate that this method can produce crystalline two-dimensional graphene platelets. The FT-IR, Raman, TGA and XPS analyses verify the successful reduction of GO even in the presence of oxidative agents and the ICP-OES analysis confirms the high purity of the final product. The characteristics of the synthesized product are comparable with other high quality chemically-synthesized RGOs.

## Methods

### Synthesis of ARGO

In a typical procedure, 1 g of graphite flakes (99%, Alfa Aesar) was added to 50 mL concentrated sulfuric acid (98%, Merck) while stirring in an ice-water bath. 3 g potassium permanganate (>99%, Sigma Aldrich) was gradually added by maintaining the temperature under 10 °C. Then, the suspension was stirred at room temperature for 25 min followed by 5 min sonication in an ultrasonic bath (SO-TEC, MUY-4). After repeating the stirring-sonication process for 12 times, the reaction was quenched by the addition of 200 mL distilled water. An extra 2h ultrasonic treatment was carried out before dividing the suspension into two equal parts; one washed to obtain AGO (described later) and the other was further processed for preparation of ARGO. After adjusting the pH at ~6 by the addition of 1M sodium hydroxide (>98%, Sigma Aldrich) solution, the suspension was further sonicated for 1 h. 10 g L-ascorbic acid (99%, Sigma Aldrich) was dissolved in 100 mL distilled water and then was slowly added to the exfoliated graphite oxide suspension at room temperature. The reduction was performed at 95 °C for 1 h. The resultant black precipitates were simply filtered by cellulose filter paper and further were washed with a 1M hydrochloric acid solution (37%, Merck) and distilled water to neutral pH. Finally, the filtrate was freeze-dried to obtain ARGO powder.

### Synthesis of AGO

The washing of AGO was adapted from a modified Hummers method^32^. 20 mL hydrogen peroxide (30%, Merck) was added to the exfoliated graphite oxide suspension and stirred until gas evolution ceased. The AGO was washed with 1M hydrochloric acid solution and distilled water for several times each for 30 min by means of centrifugation (Ependorf, 5810-R) at 10000 rpm. The final AGO precipitates were dried at room temperature.

### Instrumentation

SEM images were taken by a Leo Supra 35VP field emission scanning electron microscope. A JEOL JEM 2100F transmission electron microscope was used for TEM imaging, acquiring SAED patterns, and HR-TEM analysis. XRD patterns were recorded with a Bruker AXS advance powder diffractometer equipped with a Siemens X-ray gun, using Cu K_α_ radiation (λ = 1.5406 Å). The investigation of thermal properties was carried out using TGA (DTG-60H, Shimadzu) under air with a ramp rate of 1 °C min^−1^. Raman spectroscopy measurements were taken using a Renishaw InVia Reflex Raman microscopy system with a 532 nm laser. FT-IR spectra were collected using a Bruker Tensor 27 instrument. The XPS spectra were recorded using a VG Scientific-ESCA Lab 250 XPS spectrometer with a monochromatic Al K_α_ radiation source (1486.8 eV). The Spectral Data Processor (SDP, V.4.1) software was employed for curve fittings and atomic percent calculations of XPS spectra. The ICP-OES tests were performed using a Varian Vista-Pro Axial. The ICP-OES samples were digested in 2 mL 35% H_2_O_2_ and 4 mL 65% HNO_3_ using a microwave digestion system (MarsXpress, CEM Corp.).

## Author Contributions

S.A. and H.A. conceived and designed the research. S.A. and H.S.K. carried out the experiments. S.A. and H.A. interpreted the results and wrote the manuscript. All authors reviewed and approved the final version of the manuscript.

## Additional Information

**How to cite this article**: Abdolhosseinzadeh, S. *et al.* Fast and fully-scalable synthesis of reduced graphene oxide. *Sci. Rep.* **5**, 10160; doi: 10.1038/srep10160 (2015).

## Figures and Tables

**Figure 1:**
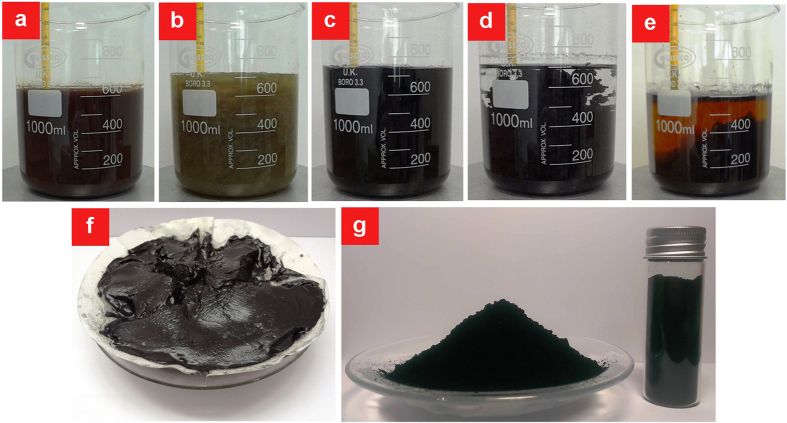
Consecutive steps of ARGO production. **a** oxidation and exfoliation of graphite in the presence of oxidizing agents. **b** reduction and conversion of insoluble manganese ions to soluble ions by addition of ascorbic acid. **c** changing the color of exfoliated graphite oxide from greenish yellow to black at the early stages of reduction. **d** loss of the hydrophilicity of GO by pausing the stirring. **e** precipitation of ARGO after accomplishment of the reduction stage and cooling down to room temperature. **f** easy filtration of ARGO by conventional cellulose filter paper. **g** attaining ARGO in the powder form after freeze drying.

**Table 1:** ICP-OES analysis results.

	**Mg**	**Zn**	**Mn**	**Fe**	**Cu**	**Ca**	**Na**	**K**	**Al**
Graphite	61.38	21.62	7.47	689.40	<detection limit	134.90	102.31	162.07	104.55
AGO	14.61	7.94	13.33	24.28	7.51	19.24	9.19	15.36	11.03
ARGO	3.98	4.91	9.92	18.60	8.49	8.69	5.20	6.97	6.12

Metallic impurities content (ppm) of the starting graphite, AGO and ARGO.
